# Special Issue: Probiotic Potential of Isolated Cultures from Spontaneously or Naturally Fermented Food Products

**DOI:** 10.3390/foods13121817

**Published:** 2024-06-09

**Authors:** Anthony N. Mutukumira, Svetoslav Dimitrov Todorov

**Affiliations:** 1 School of Food and Advanced Technology, Massey University, Auckland 0745, New Zealand; tony.mutukumira@gmail.com; 2 ProBacLab, Laboratório de Microbiologia de Alimentos, Departamento de Alimentos e Nutrição Experimental, Faculdade de Ciências Farmacêuticas, Universidade de São Paulo, São Paulo 05508-000, SP, Brazil; 3 CISAS—Center for Research and Development in Agrifood Systems and Sustainability, Instituto Politécnico de Viana do Castelo, 4900-347 Viana do Castelo, Portugal

Fermentation is probably the oldest ancient tradition used by indigenous inhabitants for the preservation of food [1]. Thus, the production of fermented food products can be traced to the earliest civilizations and can be associated with humans before the term civilization could be defined. The traditional practice was used to transform raw materials into a variety of products with unique sensory and physical characteristics, as well as extended shelf-life. In the early days, fermentation was a simple way to preserve food when in excess which was then used during the off season to improve household food security. The household practice relied on spontaneous fermentation by the inherent microorganisms in the food and environment. Traditional fermented foods were highly appreciated mainly for their distinct sensory profiles and nutritional properties. With the evolution and modernization of societies, gastronomic properties gained a protagonist role in the production, distribution, and consumption of fermented food products. However, from the perspective of the 21st century, the products may be classified as sources of highly potent and effective beneficial (probiotic) organisms and postbiotics [2]. The evolution of fermented foods has mainly depended on the type of fermenting microorganisms; moreover, knowledge on their specificity and beneficial properties can be extended to their applications not only as foods, but as health and wellbeing supplements [3]. It is certain that spontaneous fermentation gave birth to modern commercial fermentation with the development of starter cultures and probiotics with defined characteristics. Fermented foods were described as functional products from early fermentations due to the health benefits derived from numerous nutrients and microorganisms with probiotic functionality and naturally delivered to consumers as part of their nutritional diet [4]. Thus, the demand for fermented foods has increased rapidly in the last several decades due to these numerous beneficial properties. Recent advances in scientific research have shown that probiotic microorganisms may play a central role in human and animal health through their metabolism in the gastrointestinal tract (GIT) [4]. The FAO/WHO recommends the consumption of food containing at least 10^6^ live cells of probiotic microorganisms per gram or milliliter of product per day [5]. Further, the advent of plant-based, vegan, and vegetarian products has created new opportunities for the development of suitable products with probiotics for the consumers of these products. Therefore, there is an ever-increasing demand for functional food products containing probiotics that suit modern segments of our society.

This Special Issue aimed to disseminate research on the isolation and characterization of potential probiotics from a range of spontaneously fermented foods, with a view to establishing a link between microbial populations of fermented foods and health benefits for humans and animals, or, simply, to realize Hippocrates’ dream that food should be a medicine and medicine a food.

In this Special Issue of *Foods*, different papers covering a variety of topics, from microbial population of kombucha [Contribution 1], GABA (gamma-aminobutyric acid) production by *Levilactobacillus brevis* strains isolated from lichi juice [Contribution 2], beneficial effects of supplementing fermented milk with exotic fruits pulps [Contribution 3], role of probiotic oxalate-degrading bacteria in improving food quality [Contribution 4], to the role of bacteriocins and their producers in food safety of fermented food products [Contribution 5], were presented. The participation of research teams from New Zealand, China, Brazil, Russia, the USA, Iran, and Poland reflected the global interest in building the research collection.

Fermentation dates to ancient times, when it was the principal way for the biopreservation of food products. From the perspective of modern science, fermentation involves different processes, during which metabolites with antimicrobial properties are produced by different microbial species.

Consumption of tea is part of the gastronomic and cultural habits of different ethnic groups. Originally associated with the Asian culture, kombucha is gaining popularity around the world. Kombucha can be described as a sparkling sweetened tea, fermented by a symbiotic culture of acetic acid bacteria (AAB) and yeast. The popularity of kombucha continues to increase worldwide, associated not only with appealing sensory properties but also to the perceived health benefits. In the study by Wang et al. [Contribution 1], the authors reported on the isolation and characterization of the predominant AAB and yeast from a starter culture and kombucha broth during fermentation for 14 days at 22 °C. The authors have built a collection of yeast and AAB using glucose yeast extract mannitol ethanol acetic acid (GYMEA) and yeast extract glucose chloramphenicol (YGC) media, which are ideal for the isolation of the respective fermenting microorganisms. The identification of selected cultures (AAB and yeasts) was performed based on morphological and biochemical characterization, followed by a sequence analysis of the ribosomal RNA gene (16S rRNA for AAB and ITS for yeast). It was an interesting note that Wang et al. [Contribution 1] followed the fermentation for changes in the microbial composition, associated with variations in the physico-chemical characteristics of kombucha tea, such as pH, titratable acidity, and total soluble solids (TSSs). During fermentation, acidity increased and TSS decreased. The yield, moisture content, and water activity of the cellulosic pellicles which had developed at the end of fermentation were attributed to the presence of AAB. The authors pointed out that the dominant AAB species in the cellulosic pellicles and kombucha broth was *Komagataeibacter rhaeticus*. Meanwhile, predominant yeast isolates were identified to belong to *Debaryomyces prosopidis* and *Zygosaccharomyces lentus* species [Contribution 1].

The selection of appropriate fermenting microbes is an important process in further applications for exploring beneficial properties. In general, it is recommended that microbial cultures isolated from specific fermented food products are appropriately applied in similar products [3]. This is a logical choice since the microbes are part of the microbiota of the specific product which can be easily adapted to the desired environmental conditions. Similar arguments are valid when selecting probiotics from human origin. However, experimental studies clearly show that microbial cultures from different origins can be applied to fermentation processes or as probiotics, when they are suitable from a technological point of view, than to the provision of health benefits [6]. 

The study of Jin et al. [Contribution 2] isolated *Levilactobacillus* (*Lvb*.) *brevis* strains from traditional Chinese pickles. The strains were added as starter cultures to improve the nutritional profiles of fermented juices. The authors reported on three *Lvb. brevis* strains (LBG-29, LBG-24, LBD–14) with the ability to produce high levels of GABA (>300 mg/L). Upon evaluation of the technological properties, the study showed that four strains had low tolerance at a low pH and when subjected to high bile salts that were used to test the safety of the cultures in vitro. *Lvb. brevis* strains were used for the fermentation of litchi juice at 37 °C for 48 h. The litchi juice was determined to be a good substrate for fermentation as the process enhanced its functional profile. The results showed that the *Lvb. brevis* strains were well adapted to the studied environment and reached levels of >8.7 log10 CFU/mL. Further, the antioxidant activities of 2,2-diphenyl-1-picrylhydrazyl (DPPH) and ferric ion-reducing antioxidant power (FRAP) were significantly increased (*p* < 0.05), and the antioxidant capacity of the 2,20-amino-di (3-ethyl-benzothiazoline sulphonic acid-6) ammonium salt (ABTS) was decreased. A significant (*p* < 0.05) increase in GABA and acetic acid content after fermentation by LBG-29 and LBG-24 was reported. It was thus determined that the LBG-29 and LBG-24 strains may be used to improve the functionality of the beverage and aid in the development of new products. According to Jin et al. [Contribution 2], this was the first report of litchi fermentation using *Lvb. brevis* strains as starter cultures.

Performance of the beneficial microbes may be associated with the role of different environmental factors, including specificity of the diet [7]. In this regard, the role of the fruit pulps (buriti and passion fruits) supplemented in fermented milk for the modulation of intestinal microbiota was evaluated by Borgonovi et al. [Contribution 3]. In the experimental plan, the authors evaluated the effect of putative probiotic fermented milk (FM) with buriti pulp (FMB), passion fruit pulp (FMPF), and without fruit pulp (FMC) (control) on the microbiota of healthy humans in a model system (SHIME^®^), where the viability of lactic acid bacteria (LAB), microbiota composition, presence of short-chain fatty acids (SCFA), and ammonium ions were determined. The results showed that the viability of the applied starter cultures in the fermented milk was affected by the addition of the fruit pulps. The authors reported that Phocaeicola was predominant in the FMPF and FMB samples; *Bifidobacterium* was related to FM formulations, while Alistipes was associated with FMPF and FMB, and *Lactobacillus* and *Lacticaseibacillus* were predominant in FMC. Trabulsiella was the dominant element in the FMC, while Mediterraneibacter was dominant in the FMPF and FMB networks [Contribution 3]. Further, acetic acid increased in FM formulations, and high amounts of propionic and butyric acids were observed in the FMB treatment. The authors reported decreased ammonium ions compared to the control in all the FM formulations. The FMPF samples had significantly (*p* < 0.05) lower amounts of ammonia [Contribution 3]. Borgonovi et al. [Contribution 3] suggested that in addition to potential gastronomic benefits, fermented milk supplemented with buruti or passion fruit pulp may improve the beneficial effects of the intestinal microbiota by increasing the levels of SCFA and decreasing ammonium ions, which could be related to the presence of bioactive compounds.

Beneficial microbes can play an important role in the reduction in specific metabolites. Previous studies have suggested that selected microbial cultures can improve the detoxication of aflatoxins, heavy metals, and pesticides [8]. In these processes, probiotics can degrade the toxins to safe organic metabolites or adsorb them and then remove them from the human (or other animals) body [8,9]. Oxalates are produced by different edible plants; however, when digested by mammals, they can accumulate in the liver, and as a consequence, they compromise human (and other animals) health. Humans and other animals possess enzymatic systems that can degrade oxalate [Contribution 4]. However, in addition, numerous oxalate-degrading bacteria colonize the mammalian gut and can act as additional mechanisms to improve the processes of reducing the negative effects of oxalates to the host. Karamad et al. [Contribution 4] reviewed the role of the environmental factors that can influence the efficacy of probiotic oxalate-degrading bacteria. The mechanisms of oxalate catabolism by obligate and facultative anaerobic oxalate-degrading bacteria, both in vitro and in vivo environments, were discussed.

The application of bacteriocinogenic bacterial species (including LAB and *Bacillus* spp.) has been increasing in recent decades, thereby extending the traditional biopreservation processes to applications in human and veterinary medicine [Contribution 5]. Several bacteriocinogenic strains studied in the last few decades were isolated from different fermented food products from around the world [10]. The aim of the review by Todorov et al. [Contribution 5] was to thoroughly re-examine the different research methodologies used in studies focusing on the use of beneficial microbes and bacteriocins as potent biopreservatives within the food sector. A significant volume of the literature has been dedicated to the discovery of new bacterial strains from fermented foods and their potential as probiotics, which included an initial analysis of bacteriocins synthesized by these bacteria. Most of these investigations were conducted according to established standards recognized by the scientific community. The researchers suggested a range of uses for the examined strains and bacteriocins as viable biopreservatives in food production. Some studies extended their scope by conducting experimental research, assessing the integration of bacteriocins into specific food products, or determining the efficacy of the proposed probiotics and bacteriocins in combating foodborne pathogens. Certain researchers recommended the use of bacteriocin-producing cultures as initial cultures, investigating the direct production of bacteriocins to effectively manage foodborne pathogens. Nonetheless, there have been limited studies concerning the potential negative impacts of bacteriocins, such as their toxicity. This concern arises from extensive documentation indicating that bacteriocins are generally non-immunogenic and exhibit minimal cytotoxicity, attributed to the fact that these protein-based entities are typically small peptides. Yet, there have been instances where bacteriocins have demonstrated significant cytotoxic effects, which could be exacerbated in the case of genetically altered bacteriocins. Additionally, the toxicity levels of bacteriocins can vary greatly, depending on its concentration and the type of cell targeted. These aspects were elaborated upon in a review of Todorov et al. [Contribution 5].

Fermented products play an important role in the traditional diets of many consumers around the world. The evolution of these products has been dependent on the type of raw materials, fermenting microorganisms (commonly called starter cultures), and other environmental factors. The fermentation process can be triggered by inherent natural microorganisms present in the food and environment, or it can be a result of the deliberate addition of suitable cultures. Apart from fermenting the raw materials, the microorganisms or starter cultures can confer health benefits to the consumer when consumed in sufficient amounts. Thus, fermented foods are now widely known as functional products. During fermentation, starter cultures produce inhibitory organic compounds against pathogens for consumer safety and also prevent spoilage. Typical inhibitory organic compounds include the non-volatile and volatile organic acids. Further, some cultures produce bacteriocins that can have effective inhibitory compounds against pathogens. The production of bacteriocins can be optimized and applied in other food processes such as cheese-making and baking. Hence, there is a need to search for novel cultures which may have excellent fermenting properties and/or probiotic function.
